# Synthetic cartilage implant vs. first metatarsophalangeal arthrodesis for the treatment of hallux rigidus

**DOI:** 10.1007/s00402-024-05534-9

**Published:** 2024-09-17

**Authors:** Konrad Budde, Leif Claassen, Christian Plaass, Christina Stukenborg-Colsman, Kiriakos Daniilidis, Daiwei Yao

**Affiliations:** 1 Orthopedic Department, Hannover Medical School, Carl-Neuberg-Straße 1, 30625 Hannover, Germany; 2 Rueckenprofis Hannover, Luisenstraße 10-11, 30159 Hannover, Germany; 3 OTC Regensburg, Paracelcusstraße 2, 93053 Regensburg, Germany; 4 Emma Klinik, Klinik für operative Medizin GmbH & Co. KG, Frankfurter Straße 51, 63500 Seligenstadt, Germany

**Keywords:** Hallux rigidus, Metatarsophalangeal joint, Fusion, Arthrodesis, Cartiva

## Abstract

**Background:**

This study evaluated the outcome of the Cartiva synthetic cartilage implant (SCI) in the treatment of hallux rigidus.

**Methods:**

In the present retrospective matched case-control study, we compared 18 patients with Cartiva SCI (mean follow-up: 17.7 months) to 18 patients with metatarsophalangeal joint arthrodesis (mean follow-up: 20 months) using multiple function measures, along with four specified visual analog subscales for pain. Pre- and postoperative radiographs were compared, and radiographic abnormalities were documented.

**Results:**

We observed no significant differences in function measures between groups. While both groups experienced significant pain reduction, the arthrodesis group reported significantly lower exertion pain than the Cartiva SCI group (*p* = 0.004). Radiographic abnormalities, including implant site enlargement (6/18, 33.3%), erosive changes of the metatarsal bone (11/18, 61.1%) or articular surfaces (10/18, 55.6%), and bright sclerotic margins (12/18, 66.7%), occurred in the Cartiva SCI group.

**Conclusion:**

The present study showed good functional results and a high satisfaction rate after MTP joint arthrodesis, which is considered the gold standard surgical treatment for higher grade hallux rigidus. While the Cartiva SCI group did not show significant differences from the arthrodesis in most aspects of function and clinical scores, the arthrodesis group tended to have better results in terms of satisfaction, residual pain, and revision rate. Even after the short follow-up period, there were some remarkable radiographic findings in the Cartiva SCI group, the long-term effects of which are not yet evident, but which may lead to implant loss. Cartiva SCI has advantages for patients who prioritize postoperative mobility, but the potential risks should be considered in the patient’s informed consent. Therefore, the present study highlights the importance of MTP joint arthrodesis for the treatment of hallux rigidus.

**Level of evidence:**

Level IV – Retrospective matched case-control study.

## Introduction

Hallux rigidus is the second most common pathological condition of the forefoot [1]. Individuals with hallux rigidus mostly suffer from moderate to severe joint pain, and as the cartilage degeneration progresses, they may experience stiffness of the great toe with limitations in dorsiflexion and plantarflexion, loss of function, and change of gait [1, 2]. 

In the early stages of hallux rigidus, symptoms can be satisfactorily alleviated with conservative, non-surgical measures, such as flat, stiff-soled, big-toe box shoes, insoles, and anti-inflammatory injections [3]. If conservative therapy is ineffective or osteoarthritis progresses, joint-preserving procedures, such as cheilectomy or various joint-line extending osteotomies can provide relief [4]. If arthritis is already severe or end-stage, the gold standard operation is metatarsophalangeal (MTP) joint arthrodesis [4, 5]. However, a serious disadvantage of arthrodesis is the loss of first MTP joint range of motion. Total prosthetic replacement with silastic or metal implants or hemiarthroplasty showed good initial results in maintaining mobility, but it often had a high rate of fracture, loosening, instability, subsidence, radiological abnormalities, and foreign body reactions [6]. 

The Cartiva^®^ synthetic cartilage implant (Cartiva SCI, Stryker Inc., Kalamazoo, MI), a synthetic hydrogel polymer consisting of 40% polyvinyl alcohol and 60% saline, shows physical and mechanical properties similar to those of native cartilage, particularly in terms of water content and friction value [7]. Furthermore, the biocompatibility of polyvinyl alcohol implants (PVI) with surrounding tissues, such as muscle, bone, cartilage, and synovium has been described, which suggests their suitability as an implant for hemiarthroplasty [8]. The present study aimed to compare the clinical and radiological results after Cartiva SCI implantation to conventional MTP joint arthrodesis and to the inhomogeneous literature.

## Methods

After approval by the ethics committee, potential study participants were selected. We aimed to recruit all patients aged 18–80 years who underwent synthetic cartilage implantation surgery at our hospital. The indication for this procedure was moderate to severe hallux rigidus of grades 2–4, according to Kellgren and Lawrence, without the additional presence of hallux valgus [9]. For the control group, we included patients aged 18–80 years diagnosed with grades 2–4 hallux rigidus alone or in combination with mild to severe hallux valgus who were subsequently treated with MTP joint arthrodesis. The procedures were performed by 5 surgeons specializing in foot and ankle surgery with more than 6 years of experience and board certification by the national Foot and Ankle Society. Care was taken to ensure that the procedures in both groups occurred at similar time points to produce a comparable follow-up period. The decision as to which patients should undergo Cartiva SCI implantation or arthrodesis was based on individual considerations, primarily the patient’s activity profile. After a detailed information on the benefits and risks, the final decision was left to the patient.

In the main study group (Cartiva SCI), 24 patients underwent SCI hemiarthroplasty surgery between March 2018 and February 2019. Three patients could not be reached; one individual did not want to participate in the study; and two patients received SCI in the second ray and, accordingly, could not be used for further comparisons. In the end a total of 18 patients (18 feet, 14 females, 4 males, mean age: 51.7 years (range: 36–71), mean body mass index [BMI]: 26.9 kg/m^2^ (range 20.6–35.4), mean follow-up: 17.7 months (range: 12–23)) were included.

**Fig. 1:**
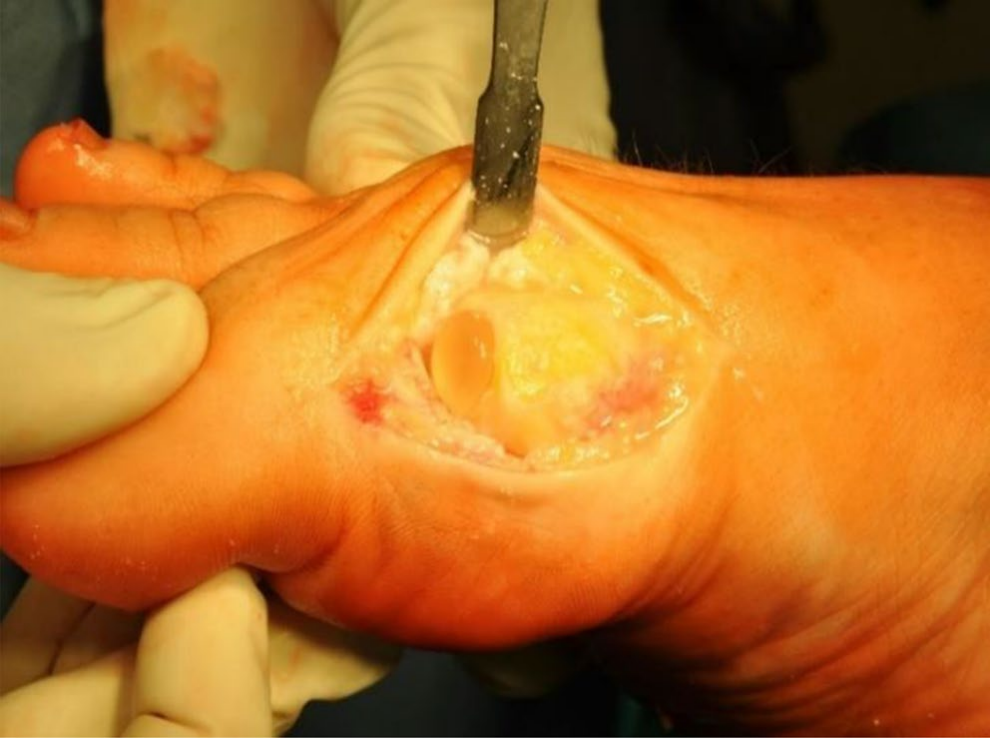
Intraoperative situation after placement of the implant in the implant site, the approximately 2 mm elevated position against the surrounding tissue is clearly visible

In all patients with Cartiva SCI, the standardized surgical technique was applied according to the manufacturer’s instructions and as described in detail by other authors [2, 10, 11]. First, the MTP joint was made accessible through a straight mediodorsal incision. Then, all existing osteophytes were removed dorsally, medially, and laterally, paying special attention to preserve the cortical rim of the MTP head. The implant site was visualized by flexion of the proximal phalanx, followed by preparation of an implant site adequate for the selected size of the Cartiva implant using a guidewire and a step drill. All included patients were treated with the largest available polyvinyl alcohol hydrogel implant (10 × 10 mm). For proper function, an implant position 1–2 mm above the surrounding bone and cartilage was used (Fig. 1). After placement of the implant and control of function by passive movements intraoperatively, the joint capsule and, subsequently, the skin were sutured. The last step was the final control of mobility. Postoperatively, the patients were instructed to wear a hard-sole shoe for six weeks. Full weight bearing was permitted and desired after a few days. In addition, the patients received mobilization exercise instruction, and physiotherapy was prescribed.

In the control group, 61 patients, who underwent a total of 64 arthrodeses between March 2018 and November 2019, were identified. The arthrodeses were performed with a crossing lag screw and an additional dorsal locking plate. Postoperatively, patients were required to wear a bandage shoe for 6 weeks. To increase the comparability between groups regarding the characteristic parameters of preoperative hallux rigidus grade, age, sex, and BMI, a propensity score-based 1:1 matching between groups was conducted (package MatchIt for R, nearest neighbor method). This method identified 18 patients in the control group who were used for further analysis (18 feet, 15 females, 3 males, mean age: 57.6 years (range: 39–77), mean body mass index [BMI]: 27.2 kg/m^2^ (range (22.0-37.5), mean follow-up: 20 months (range: 11–28)). To ensure a proper match between groups, Mann–Whitney-U tests were performed to compare the four parameters. Only the age parameter could not be matched without significant differences. Table 1 presents the demographic parameters of the study groups.

**Table 1:** Demographic parameters of both study groups

Treatment	Sex (*n*)	Age (years) Mean (Range), SD	BMI (kg/m^2^) Mean (Range), SD	Follow-up (months) Mean (Range), SD	Preoperative degree of osteoarthritis *n* (% of population)
Cartiva SCI *n* = 18	14 Females 4 Males	51.7 (36–71), 7.6	26.9 (20.6–35.4), 3.8	17.7 (12–23), 3.8	Grade 2: 5 (27.8%) Grade 3: 10 (55.6%) Grade 4: 3 (16.6%)
Arthrodesis *n* = 18 (out of 61)	15 Females 3 Males	57.6 (39–77), 8.6	27.2 (22.0-37.5), 4.5	20 (11–28) 5.4	Grade 2: 4 (22.2%) Grade 3: 7 (38.9%) Grade 4: 7 (38.9%)
*p*-value	0.791	0.024	0.913	0.214	0.293

*Abbreviations* SCI, Synthetic Cartilage Implant; n, number; BMI, body mass index; SD, standard deviation

For both groups, clinical and functional parameters were measured using the Foot and Ankle Ability Measure (FAAM), FAAM Sports, and the European Foot and Ankle Society (EFAS) score. Physical and psychological conditions were also recorded using the 36-item Short Form Survey Version 2.0 (SF-36 V2.0), which is a measure of general health status. In addition, a questionnaire that included satisfaction with the surgical outcome, willingness to undergo the same surgery again, pain-free walking distance, possible walking distance, and pre-existing conditions and diseases was completed. The questionnaire on pre-existing conditions and diseases included questions on hypertension, arthrosis, rheumatism, thrombosis, cancer, diabetes, vascular calcification, depression, varicose veins, alcohol and tobacco consumption, lung problems, hormonal contraception, osteoporosis, sensory disturbances in the legs, and circulatory disorders in the legs. Visual analog scale (VAS) scores for pain at exertion, pain at rest, pain at night, and pain at start-up movements were assessed for the preoperative and postoperative conditions in both groups. In a follow-up appointment a physical examination was performed especially regarding joint mobility in the Cartiva SCI patients, as well as the localization of pain and pressure pain, if they occurred, were measured.

To assess the condition of the joint space and any bony changes, weight-bearing anteroposterior and lateral radiographs of the forefoot were obtained as part of the routine clinical examination. Preoperative radiographs were available for all patients in both groups. Differences between the preoperative and postoperative characteristics were measured, and radiographic abnormalities were documented.

### Statistical analysis

Normal distribution was tested using the Shapiro–Wilk test. The outcome parameters were tested within (Wilcoxon test: preoperative vs. postoperative) and between (Mann–Whitney U test: Cartiva SCI vs. MTP joint arthrodesis) the study groups. A threshold for statistical significance (*P* = 0.05) was set for all comparisons. A Pearson Chi-Square test was used to analyse correlations between sex and pre-existing conditions and diseases. Statistical analysis was conducted via SPSS (Version 26, IBM Corp, Armonk, NY).

## Results

### Functional and clinical outcomes

The postoperative FAAM, FAAM Sports, EFAS, and SF-36 scores showed no significant differences between the groups (Table 2). While the FAAM (Mean: Cartiva SCI 83.4 vs. MTP joint arthrodesis 82.3, *p* = 0.584) and SF-36 (Physical: 50.1 vs. 48.0; *p* = 0.938, Psychological: 50.9 vs. 49.9; *p* = 0.65) were almost identical, the FAAM Sports (65.5 vs. 71.1; *p* = 0.883) and EFAS score (15.9 vs. 19.0; *p* = 0.537) were slightly higher in the arthrodesis group than those in the Cartiva SCI group.

**Table 2:** Postoperative clinical and functional scores

	Cartiva SCI *n* = 18	Arthrodesis *n* = 18	Comparison between groups
	Mean (SD) Min-Max	Mean (SD) Min-Max	*p*-value
FAAM	83.4 (15.5) 45.2–100.0	82.3 (21.6) 13.1–100.0	0.584
FAAM Sports	65.5 (26.5) 18.8–100.0	71.1 (33.4) 6.3–100.0	0.883
EFAS Score	15.9 (6.3) 2.0–24.0	19.0 (5.3) 10.0–24.0	0.537
SF-36 Physical	50.1 (6.3) 34.8–58.6	48.0 (13.3) 16.4–63.5	0.938
SF-36 Psychological	50.9 (9.1) 29.7–62.5	49.9 (8.9) 26.5–59.5	0.65

*Abbreviations* SCI, Synthetic Cartilage Implant; n, number; SD, Standard Deviation; Min, Minimum value; Max, Maximum value; FAAM, Foot and Ankle Ability Measure; EFAS, European Foot and Ankle Society; SF-36, 36-Item Short Form Survey

Half of the patients with Cartiva SCI reported being very satisfied or satisfied with the outcome (9/18); 33.3% (6/18) were moderately satisfied; 16.7% (3/18) were dissatisfied; and none were very dissatisfied. In the MTP joint arthrodesis group, 83.3% (15/18) were very satisfied or satisfied; 16.7% (3/18) were moderately satisfied; and none were dissatisfied or very dissatisfied (Table 3). Nevertheless, in both groups, most patients would undergo their surgery a second time (Cartiva SCI: 14/16, 87.5%, two patients gave no answer; MTP joint arthrodesis: 16/18, 88.9%). The Cartiva SCI group showed only moderate, non-significant improvements in the pain-free and possible walking distances (*p* = 0.092; *p* = 0.915, respectively), whereas in arthrodesis patients, the improvements were significant (*p* = 0.001; *p* = 0.034, respectively). A direct comparison between the groups revealed a noticeable, but not significant, advantage of MTP joint arthrodesis regarding the postoperative pain-free walking distance (*p* = 0.279). However, the values for the postoperative possible walking distance were similar (*p* = 0.935, Table 4).

**Table 3:** Satisfaction with surgical outcome

		Cartiva SCI *n* = 18	Arthrodesis *n* = 18	Comparison between groups
	5-point Likert Scale	Number of patients n (%)	Number of patients n (%)	*p*-value
Satisfaction with surgical outcome	Very satisfied = 1	5 (27.8%)	8 (44.4%)	0.074
	Satisfied = 2	4 (22.2%)	7 (38.9%)	
	Moderately satisfied = 3	6 (33.3%)	3 (16.7%)	
	Dissatisfied = 4	3 (16.7%)	0	
	Very disssatisfied = 5	0	0	
	Mean, Median; Interquartile Range (Scale)	2.39, 2.5; 3.0	1.72, 2.0; 1.0	

**Table 4:** Outcome parameters regarding pain-free and possible walking distance

		Cartiva SCI *n* = 18			Arthrodesis *n* = 18			Comparison between groups	
		Preoperative	Postoperative	Pre- vs. postoperative	Preoperative	Postoperative	Pre- vs. postoperative	Preoperative	Postoperative
	Scale 0–3	Number of patients		*p*-value	Number of patients		*p*-value	*p*-value	*p*-value
Pain-free walking distance	< 0.5 km = 3	9	5	0.092	7	1	0.001	0.782	0.279
	0.5–2 km = 2	4	3		5	1			
	2–3 km = 1	2	0		3	4			
	> 3 km = 0	3	10		2	12			
	Mean (Scale)	2.06	1.17		2.00	0.50			
Possible walking distance	< 0.5 km = 3	2	2	0.915	3	1	0.034	0.343	0.935
	0.5–2 km = 2	2	1		2	0			
	2–3 km = 1	2	3		3	5			
	> 3 km = 0	12	12		7	11			
	Mean (Scale)	0.67	0.61		1.07	0.47			

Between the preoperative and postoperative conditions, there was a significant improvement in pain on all VAS subscales in both groups. While the preoperative pain scores were higher in the arthrodesis patients than in the Cartiva SCI patients regarding all subscales, their mean postoperative scores were lower than those of the Cartiva SCI patients. For pain at exertion, a significant correlation was detected (*p* = 0.004), but a trend was also evident in the other subscales (Table 5). Ten Cartiva patients were able to localize their pain to the MTP joint; three patients suffered metatarsalgia in the second forefoot ray and two patients in the third forefoot ray, while two patients described pain in the area of the sesamoids. Only two Cartiva patients were completely pain-free. Pressure pain occurred in 11/18 (61.1%) Cartiva patients and was found exclusively in the MTP head, MTP joint, proximal phalanx, or lateral sesamoid. In addition, there was a pressure dolent MTP-III head.

**Table 5:** Outcome parameters regarding pain

	Cartiva SCI *n* = 18			Arthrodesis *n* = 18			Comparison between groups	
	Preoperative	Postoperative	Pre- vs. postoperative	Preoperative	Postoperative	Pre- vs. postoperative	Preoperative	Postoperative
VAS 0–100	Mean (SD) Min-Max	Mean (SD) Min-Max	*p*-value	Mean (SD) Min-Max	Mean (SD) Min-Max	*p*-value	*p*-value	*p*-value
Pain on exertion	69.9 (18.0) 33.0–97.0	35.4 (25.7) 0.0–93.0	0.002	71.9 (14.4) 40.0–93.0	11.8 (14.6) 0.0–49.0	< 0.001	0.839	0.004
Pain at rest	37.3 (32.3) 0.0–94.0	12.1 (18.2) 0.0–60.0	0.004	48.3 (29.1) 0.0–91.0	3.4 (8.3) 0.0–35.0	< 0.001	0.265	0.171
Pain at night	24.1 (32.7) 0.0–89.0	3.8 (7.0) 0.0–29.0	0.007	28.9 (23.6) 0.0–94.0	1.9 (3.5) 0.0–12.0	0.001	0.195	0.406
Pain on start-up	48.3 (30.7) 0.0–99.0	16.4 (17.8) 0.0–63.0	0.003	56.1 (30.0) 3.0–95.0	5.3 (6.9) 0.0–20.0	< 0.001	0.462	0.059

*Abbreviations* SCI, Synthetic Cartilage Implant; n, number; VAS, Visual Analog Scale; vs., versus; SD, Standard Deviation; Min, Minimum value; Max, Maximum value

The maximum range of motion compared to the preoperative to postoperative state was only of concern for the Cartiva SCI group. There was a significant decrease in the maximum possible dorsiflexion from a mean of 32.5 (range: 10–70) degrees preoperatively to 21.1 (range: 5–40) degrees postoperatively (*p* = 0.013), whereas the maximum plantarflexion increased slightly from 16.0 (range: 0–30) to 18.6 (range: 5–30) degrees (*p* = 0.526).

The Pearson-Chi-Square test, which examines the associations between sex and pre-existing conditions and diseases, showed no correlations.

### Radiological outcome

**Fig. 2:**
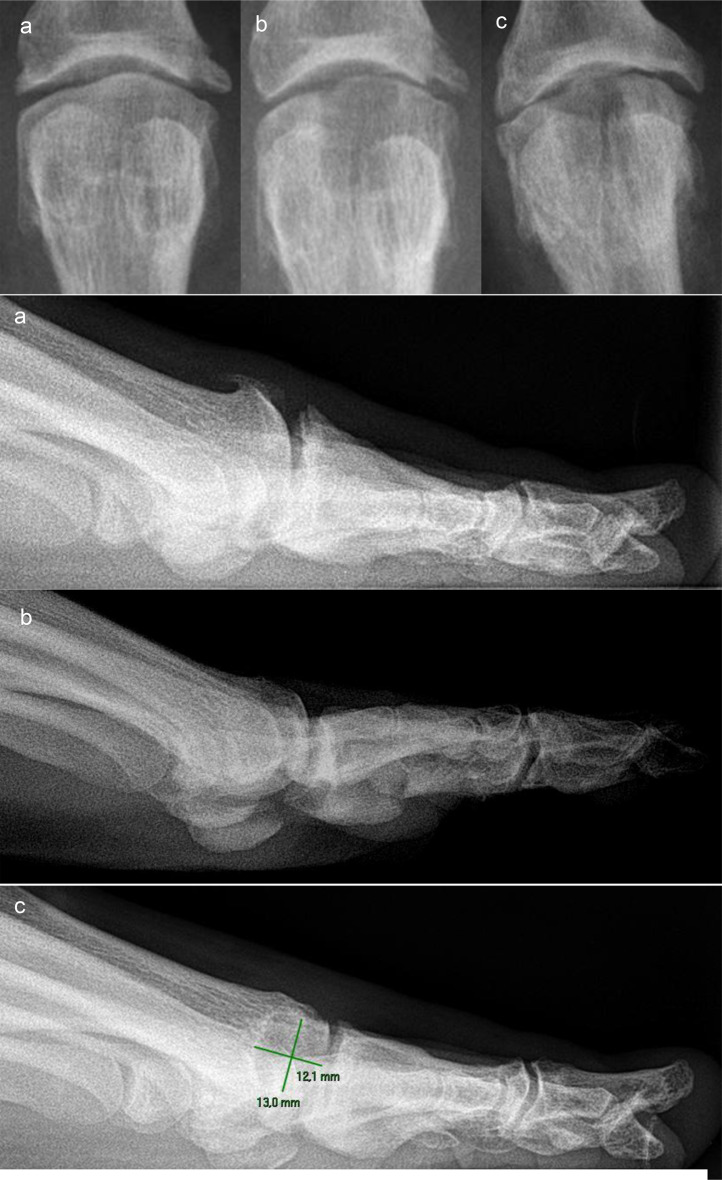
Case of a satisfactory clinical outcome **a**: Preoperative condition. **b**: Radiological control 6 weeks postoperatively: no abnormalities and satisfactory joint space. **c**: Follow-up, 23 months postoperatively: Joint space now reduced to some considerable extent, but mobility satisfactory. No reaction of the surrounding tissue in the anteroposterior projection, but enlargement of the implant site in the lateral radiograph. The patient is completely pain-free

**Fig. 3:**
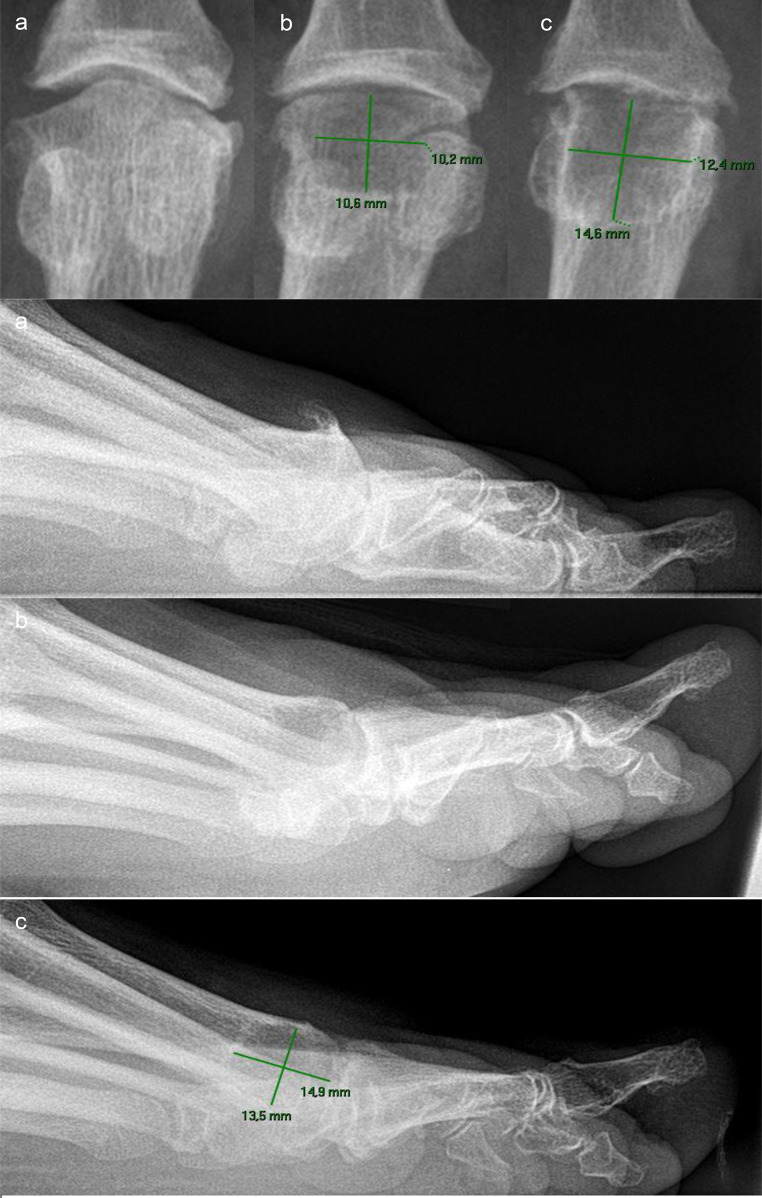
Severe case of osteolytic expansion of the implant site **a**: Preoperative condition. **b**: Radiographic control 2 days postoperatively: correct location of the bone canal of adequate size for a 10 × 10 mm Cartiva SCI. **c**: Follow-up, 13 months postoperatively: Extensive enlargement of the implant site to 12.4 × 14.6 mm in anteroposterior projection respectively 13.5 × 14.9 mm in lateral projection. Additionally: bright sclerotic margin, severe erosive changes on the bone surrounding the implant and the articular surface of the proximal phalanx

**Fig. 4:**
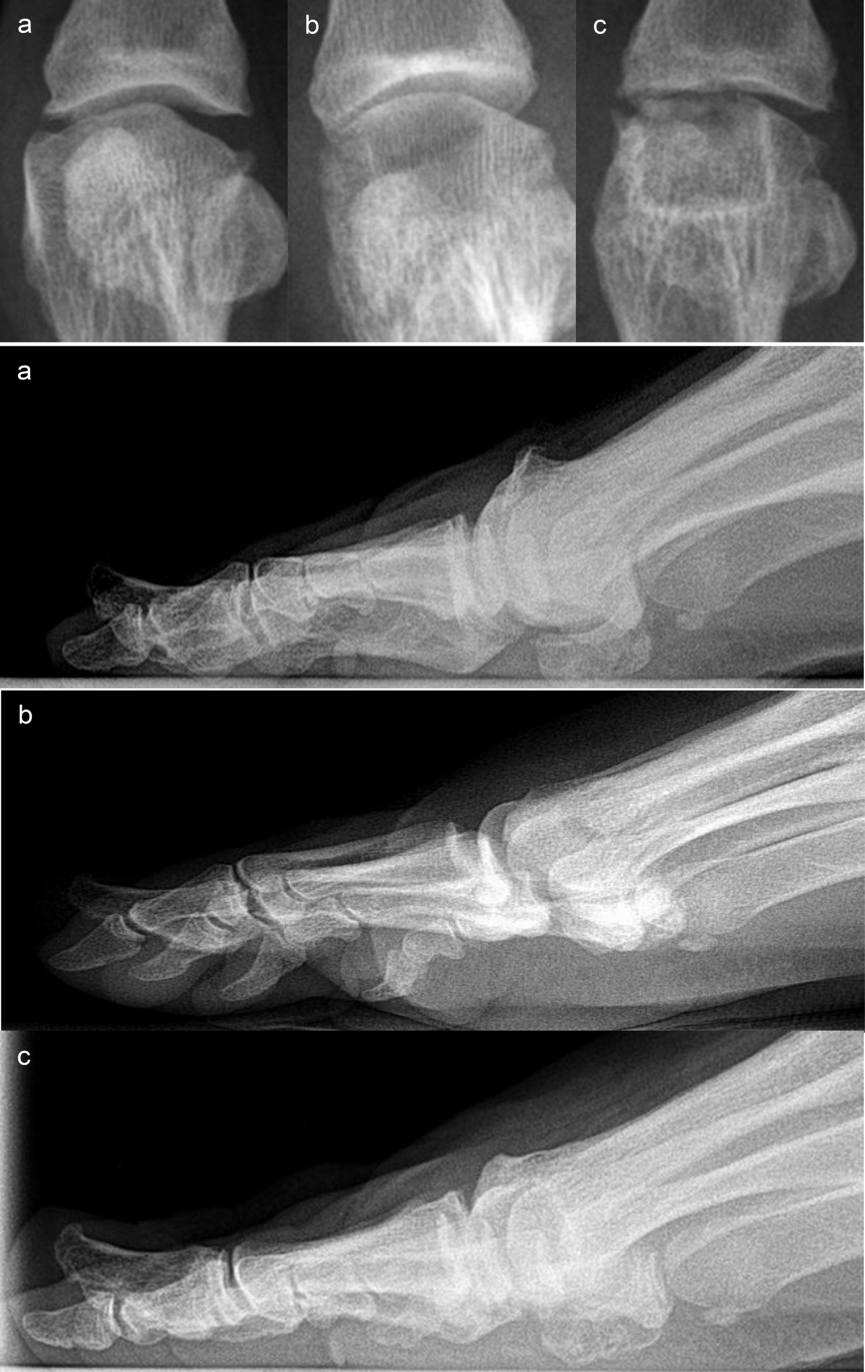
Case with pronounced bright sclerotic margin around the implant **a**: Preoperative condition. **b**: Postoperative control radiograph: Regular position of the bone canal **c**: Follow-up at 21 months postoperatively, severe bright sclerotic margin and severe erosive changes on the bone surrounding the implant and the articular surface of the proximal Os metatarsale I

The radiographic evaluation revealed some abnormalities in the Cartiva SCI group. Bony changes occurred in 11/18 patients (61.1%), joint space narrowing in 10/18 patients (55.6%), and bright sclerotic margins in 12/18 patients (66.7%). 10/18 patients (55.6%) had erosions of the articular surfaces, 11/18 (61.1%) had erosions of the metatarsal bone in the area surrounding the implant, and 6/18 (33.3%) had partially extensive enlargement of the implant site by several millimeters in both width and depth (Table 6). Figures 2, 3 and 4 show examples of the specific radiographic abnormalities.

**Table 6:** Overview of the occurrence of various radiological abnormalities in Cartiva SCI patients

	Cartiva SCI *n* = 18					
	Osteophyte neoplasm	Joint space narrowing	Bright sclerotic margins	Erosion of articular surface	Erosion of metatarsal bone	Enlargement of implant site
Number of patients	11 (61.1%)	10 (55.6%)	12 (66.7%)	10 (55.6%)	11 (61.1%)	6 (33.3%)

In parentheses: percentage of affected patients

Revision with subsequent arthrodesis was recommended and performed in 3/18 Cartiva SCI patients (16.6%) after follow-up. One revision was performed at 23 months postoperatively due to enlargement of the implant site to 14.6 × 12.4 mm (Fig. 3) and mild persistent pain (VAS = 24), another revision was performed at 24 months postoperatively due to enlargement of the implant site to 11 × 15 mm with massive persistent pain (VAS = 93). The last revision was performed at 17 months postoperatively due to advanced erosion of the articular surfaces and persistent pain (VAS = 30).

Postoperative radiographic abnormalities were not observed in the matched control group. Bone consolidation of the arthrodesis material and bony interfaces always progressed according to stage. One arthrodesis that showed regular osseointegration was revised because of an incorrect fixation position with plantarization of the IP joint, resulting in a revision rate of 5.6%.

## Discussion

Our retrospective, matched case-control study showed a significant improvement in all pain subscales in both Cartiva SCI and arthrodesis patients when comparing preoperative to postoperative status, whereas in Cartiva SCI some mobility of the great toe could be preserved postoperatively (mean dorsiflexion 21.1° and plantarflexion 18.6°).

To establish comparability with other studies, standardized scores, especially the FAAM, FAAM Sports, and EFAS, have proven to be reliable tools for assessing foot function. Martin et al. described the FAAM and FAAM Sports as reliable, valid, and responsive measures of musculoskeletal disorders of the leg, foot, and ankle [12]. Matheny et al. also demonstrated good reliability and validity of the FAAM and FAAM Sports in patients who had undergone ankle surgery [13]. The EFAS score was evaluated by Chausse et al. and found to be suitable for assessing forefoot functional recovery after only 6 months [14]. Although there appears to be a weak correlation between the EFAS score and the SF-36 according to Chausse et al., the SF-36 is more appropriate for representing the impact of a musculoskeletal or other disease on general well-being and is not specific to foot and ankle conditions [14, 15]. However, because the SF-36 is subject to different weightings in some countries and version 2.0, which we used, is evaluated differently from version 1.0, a comparison with other authors is hardly possible here [16]. Compared to other studies, our Cartiva SCI patient population (FAAM: 83.4, FAAM Sports: 65.5) showed lower scores, for example the study by Baumhauer et al. (FAAM: 90.4 and FAAM Sports: 79.5; after 2 years) [2]. Other authors who measured FAAM and FAAM Sports for Cartiva SCI patients also tended to show higher scores than in our study, especially Daniels et al. (FAAM: 92.7, FAAM Sports: 82.3; after 2 years), Brandao et al. (FAAM: 87.0; FAAM Sports: 76.4/74.9; after a minimum of 1 year) and Engasser et al. (FAAM: 88.2, FAAM Sports: 72.0, average follow-up: 18.9 months) [17–21]. Sánchez-Guzmán et al. also summarized the favorable functional prognosis of the Cartiva SCI in a systematic review [22]. 

Our arthrodesis patients were consistently more satisfied with their outcome (83.3% very satisfied or satisfied, mean rating 1.72, median 2.0, range 1–3) than our Cartiva SCI patients (50% very satisfied or satisfied, mean rating 2.39, median 2.5, range 1–4), even though this correlation was not statistically significant (*p* = 0.074). It is conceivable that because the preserved mobility in the MTP joint regarding dorsiflexion was significantly lower than the preoperative condition (*p* = 0.014), the expectations were often not fulfilled. Considering only the scores for the PVA implant, these values were similar to those reported by Shimozono et al. (36.4% very satisfied or satisfied, mean rating 2.8 ± 1.4, range 1–5), Cassinelli et al. (42% very satisfied or satisfied, mean rating 2.9, range 1–5), and An et al. (mean rating 2.25, median 2.0), who critically discussed the outcome of PVA implants for the therapy of hallux rigidus [23–25]. In contrast, there were differences between our results and those of the Level IV study by Lee et al. (74% very satisfied or satisfied, mean rating 2.11, range 1–5) [26]. 

Often responsible for the dissatisfaction of many Cartiva SCI patients was also residual pain. Residual pain occurred primarily during walking or other physical activities, and the mean values of postoperative exertion pain in Cartiva SCI patients were significantly higher than those in arthrodesis patients (VAS: Cartiva SCI = 35.4 vs. MTP joint arthrodesis = 11.8, *p* = 0.004). The pain occurred mainly in the immediate area of the MTP joint, reinforcing the assumption of continuing progressive degenerative and inflammatory changes in the joint. The partial occurrence of pain in the MTP-II and MTP-III regions is probably due to changes in gait and associated incorrect loading of the foot. Our results differ from the two-year follow-up results of Baumhauer et al. (VAS pain = 14.5) and from the 5.8-year follow-up results of Glazebrook et al. (VAS pain < 10), both of which showed better dorsiflexion compared to baseline with almost pain-free conditions [2, 27]. There is also a big difference to the results of Daniels et al. (VAS pain = 5.7; after 5 years) [18]. Hoskins et al. did not include a VAS for residual pain in Cartiva SCI patients to their study but described the occurrence of mild pain in only 2 patients (10%), with no further complications or reoperations with an average follow-up of 30 months; in addition, dorsiflexion and plantarflexion improved significantly from preoperative to postoperative results [28]. Zanzinger et al. (VAS = 35.0) and Engasser et al. (VAS = 31.0) came to similar results as in our study, but did not seem to give this persistent pain much weight in the overall assessment of outcome [19, 29]. In contrast, Cassinelli et al. explicitly reported the impact that residual pain can have on outcome (about 50% of patients reported persistent pain and considered treatment with corticosteroid injections) [25]. Guyton et al. even set the threshold for “unacceptable” pain at VAS scores above 30 [30]. 

It is still unclear whether and to what extent patient selection according to certain parameters plays a role in the outcome of Cartiva SCI. While Goldberg et al. found no significant differences between age, gender, BMI, preoperative degree of arthritis, preoperative range of motion, preoperative pain intensity, preoperative surgeries and the outcome of Cartiva SCI, Zanzinger et al. found that patients with moderate preoperative degree of arthritis seemed to benefit most from Cartiva SCI [29, 31]. The study by Lunati et al. regarding age-dependence of functional outcome in patients with MTP joint arthrodesis suggests that there is no correlation between age and functional outcome, pain, complications, or time frame to full weight bearing [32]. Of course, these findings are not directly applicable to hemiarthroplasty with Cartiva SCI, therefore, a correlation between age or preoperative activity level and functional outcome cannot be excluded.

Within our relatively short follow-up period of 17.7 months, suspicious radiographic findings already occurred. We found a high rate of patients with radiographic evidence of erosive processes of the MTP joint surfaces (10/18, 55.6%) and metatarsal bone (11/18, 61.1%), both associated with bone and cartilage loss. The radiographs of 6/18 patients (33.3%) showed enormous enlargement of the implant sites in the bone, which promoted subsidence of the implant and may not guarantee the positional stability of the implant in the long term. The fact that the surrounding bone reacted to the implant in many cases contrasts with the good biocompatibility of polyvinyl alcohol implants postulated by Noguchi et al., who reported the occurrence of rare low-grade inflammatory reactions [8]. 

In the current literature, some authors have discussed radiographic findings in Cartiva SCI patients, with different results. Lewis et al. found radiographic abnormalities such as peri-implant lucency, implant subsidence, and osteophyte neoplasms in a study design very similar to our study, they also had a 29% rate of patients who required further surgery [33]. Shimozono et al. reported a high rate of implant subsidence with loss of joint space (in 9/10 patients), wear and volume loss of the implant, peri-implant osteolytic changes and erosive processes at the proximal phalanx [23]. Further radiological studies using magnetic resonance imaging (MRI) in symptomatic patients with persistent pain were also performed by An et al. The authors reported many cases of progression of osteoarthritis, loss of joint space with wear and subsidence of the implant, peri-implant fluid and enlargement of the implant site with implant loosening [24]. In contrast, Daniels et al. reported few radiographic abnormalities (2 cystic changes, no evidence of implant loosening or subsidence) after 5 years [18]. In follow-up period, three patients (16.7%) underwent revision surgery with subsequent arthrodesis, this loss rate is similar to that of the original patient collective reported by Glazebrook et al. after 5.8 years (15.1%) [27]. A more detailed examination using magnetic resonance imaging (MRI) would have enabled a more precise overview of the reactions of the surrounding tissue. Knowledge of the shape of the implant site, as well as the size and exact position of the implant, could provide further information about the reasons for the reactions of the tissue, such as wear of the implant, reactions to wear debris, subsidence or loosening of the implant, or other damage. The revision rate of 5.6% in the control group is consistent with the results of Marks, who reported fusion rates between 94% and 98% with high patient satisfaction, while Raikin et al. reported an average fusion rate of approximately 90% (77–100%) [34, 35]. 

One aspect that should be considered in the future is that modifications of the original surgical method could further increase the efficacy of PVA implants. As shown by Eble et al., Moberg osteotomy performed simultaneously with SCI implantation reduced pain significantly more than implantation alone, while Chrea et al. showed that Moberg osteotomy alone produced better results in terms of pain intensity and physical function than a combination of polyvinyl alcohol implant and Moberg osteotomy [36, 37]. Cassinelli et al. also advocated for additional Moberg osteotomy to improve postoperative mobility and prevent stiffness [25]. The same study showed that an elevated implant position of 2–2.5 mm above the surrounding tissue could not prevent implant subsidence. Zanzinger et al. even advocated an elevated implant position of 3 mm to prevent subsidence, but also pointed out that this can increase capsular tension, reduce mobility, increase implant loading and thus increase the risk of wear and implant loosening [29]. 

Limitations of our study are based on the retrospective design of the study and the relatively small number of Cartiva SCI patients that could be included and the short follow-up time of 17.7 (12–23) months. From a collective with a total of 61 MTP joint arthrodesis patients, 18 individuals were propensity-matched to the main group to at least minimize fluctuations in basic parameters. The lack of a priori analysis and correction for multiple hypotheses may contribute to some bias. Furthermore, it must be questioned whether surgeon-dependent parameters also had an influence on the outcome, such as a poor learning curve for the surgeons or a lack of practice in handling the material due to the small number of implants inserted. In addition, the involvement of multiple surgeons can lead to inhomogeneity in outcomes. To improve comparability and validity, prospective studies with larger patient cohorts should be planned in the future.

## Conclusion

The present study showed good functional results and a high satisfaction rate after MTP joint arthrodesis, which is considered the gold standard surgical treatment for higher grade hallux rigidus. While the Cartiva SCI group did not show significant differences from the arthrodesis in most aspects of function and clinical scores, the arthrodesis group tended to have better results in terms of satisfaction, residual pain, and revision rate. Even after the short follow-up period, there were some remarkable radiographic findings in the Cartiva SCI group, the long-term effects of which are not yet evident, but which may lead to implant loss. Cartiva SCI has advantages for patients who prioritize postoperative mobility, but the potential risks should be considered in the patient’s informed consent. Therefore, the present study highlights the importance of MTP joint arthrodesis for the treatment of hallux rigidus.

## Data Availability

The image data was extracted from the PACS of the Orthopedic Department of the Hannover Medical School. The raw data for Figures 1-4 and Tables 1-6 are not publicly available to protect individual privacy in accordance with the European Data Protection Regulation.
